# Si/Graphite@C Composite Fabricated by Electrostatic Self-Assembly and Following Thermal Treatment as an Anode Material for Lithium-Ion Battery

**DOI:** 10.3390/molecules29174108

**Published:** 2024-08-29

**Authors:** Jintao Yao, Guangzhao Zhu, Jingrui Huang, Xiaoru Meng, Maolong Hao, Shoupu Zhu, Zhen Wu, Fanxu Kong, Yue Zhou, Qi Li, Guowang Diao

**Affiliations:** 1 College of Energy Storage Technology, Shandong University of Science and Technology, Qingdao 266590, China; yaojttt@163.com (J.Y.); 15553816961@163.com (G.Z.); 19326402462@163.com (J.H.); laura2985951623@163.com (X.M.); hml11231016@163.com (M.H.); zhouyue@sdust.edu.cn (Y.Z.); 2 College of Electrical Engineering and Automation, Shandong University of Science and Technology, Qingdao 266590, China; kongxu.nachuan@foxmail.com; 3 School of Energy and Power Engineering, Jiangsu University, Zhenjiang 212013, China; 4 FEB Research Institute, Far East Battery, Wuxi 214200, China; 5 Suzhou Institute of Nano-Tech and Nano-Bionics, Chinese Academy of Sciences, Suzhou 215123, China; qli2013@sinano.ac.cn; 6 School of Chemistry and Chemical Engineering, Yangzhou University, Yangzhou 225002, China; gwdiao@yzu.edu.cn

**Keywords:** Si, graphite, lithium-ion battery

## Abstract

Commercial graphite anode has advantages such as low potential platform, high electronic conductivity, and abundant reserves. However, its theoretical capacity is only 372 mA h g^−1^. High-energy lithium-ion batteries have been a research hotspot. The Si anode has an extremely high specific capacity, but its application is hindered by defects such as large volume changes, poor electronic conductivity, and a small lithium-ion diffusion coefficient. Here, the Si/thermally reduced graphite oxide@carbon (Si/RGtO@C) composite was fabricated by electrostatic self-assembly followed by thermal treatment. The RGtO synergistic carbon coating layer can effectively compensate for the low electronic conductivity and buffer the volume expansion effect of the Si nanoparticles during charge/discharge cycles. The Si/RGtO@C anode demonstrated a significantly increased capacity compared to the RGtO. After 300 cycles, Si/RGtO@C kept a discharged capacity of 367.6 mA h g^−1^ at a high current density of 1.0 A g^−1^. The Si/RGtO@C anode shows an application potential for commercial high-energy lithium-ion batteries.

## 1. Introduction

High-energy rechargeable lithium-based batteries such as Li-air batteries [1], Li-sulfur batteries [2], and all-solid-state lithium metal batteries [3] have been a focus of research in recent years. However, there are still some unresolved challenges for the large-scale application of these batteries. The high energy density and long cycle life have led to the widespread applications of lithium-ion batteries in electric vehicles and consumer electronics [4]. However, the capacity of the graphite anode in commercial lithium-ion batteries is restricted by the theoretical maximum of 372 mA h g^−1^ [5]. To address the increasing demand for high-energy-density lithium-ion batteries, the high-theoretical-capacity anode active materials, such as transition metal oxides [6], transition metal sulfides [7], phosphorus [8], and Si [9,10,11], have been explored and researched.

Among the candidates, the Si anode has attracted much attention due to the ultrahigh theoretical capacity (4200 mA h g^−1^), low lithium insertion potential, and abundant reserves. However, Si undergoes significant volume expansion (>300%) during lithium-ion insertion which causes Si to endure excessive stress and leads to mechanical fracture and solid–electrolyte interphase (SEI) layer instability. The repetitive volume changes lead to accelerated capacity fading [12,13]. Meanwhile, the low electronic conductivity and small lithium-ion diffusion coefficient make Si exhibit an inferior charge/discharge rate performance [14]. To mitigate the problems, an effective strategy is to modify the Si anode with carbonaceous material. The Si/C anodes have been shown to obviously improve electrochemical performance [15,16,17]. Carbon materials can compensate for the low electronic conductivity of Si and mitigate the effects of volume expansion. Additionally, the carbon coating on the Si surface can improve the electronic conductivity and stabilize the SEI film [18]. The nano-Si/graphite is considered to be a promising anode material for high-energy lithium-ion batteries [19]. The interfacial contact between Si and graphite is crucial for electrochemical performance [20]. Si/graphite@C anodes often exhibit much better electrochemical performance than Si/graphite [20,21]. The carbon coating on Si improves the interfacial compatibility of Si and graphite, thus leading to a more stable structure and enhanced cycling performance [20]. Zhu et al. [21] designed a novel Si@graphite@C composite that maintained a capacity of approximately 709 mA h g^−1^ after 200 cycles at 0.2 C with a capacity retention of 86.15%. Compared to Si@graphite, the much higher capacity and enhanced cycle performance of Si@graphite@C demonstrate the beneficial role of carbon coating in promoting stable SEI formation [21]. To facilitate commercial applications, developing a mass production process for the Si/graphite@C composite is worth exploring.

In this study, Si/Graphite@C was fabricated by simple electrostatic self-assembly and subsequent thermal treatment. A commercial graphite oxide (GtO) was selected as the carrier for Si. The Si nanoparticles were modified with the cationic surfactant cetyltrimethylammonium bromide (CTAB) to impart a positive charge. The Si/GtO composite was obtained by electrostatic assembly between Si@CTAB and GtO. The Si/RGtO (reduced GtO)@C composite was fabricated by a subsequent thermal treatment using polyacrylonitrile (PAN) as the carbon source of the carbon layer. At a current density of 1.0 A g^−1^, Si/RGtO sustained a capacity of 367.6 mA h g^−1^ after 300 cycles. During the cycle, Si/RGtO exhibits high capacity retention (81.1%) and excellent cycling performance, indicating a potential as anode material for high-performance lithium-ion batteries.

## 2. Results and Discussion

### 2.1. Synthesis and Structural Characterization

**Figure 1:**
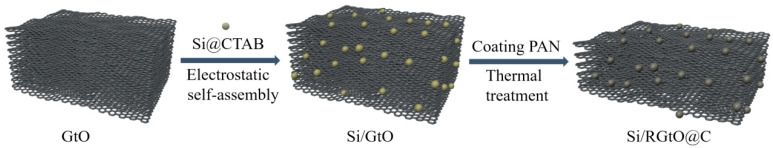
Schematic diagram of the fabrication process of Si/RGtO@C.

The preparation process of Si/RGtO@C is schematically depicted in Figure 1. Si/GtO was obtained by the electrostatic self-assembly between positively charged Si@CTAB and GtO in dispersion. Si/RGtO@C was fabricated by thermal treatment of the Si/GtO composite of coating PAN at 250 °C in an air atmosphere, followed by treatment at 800 °C in an Ar atmosphere. During this process, the coating PAN was transformed into a carbon coating layer and GtO was reduced to RGtO.

**Figure 2:**
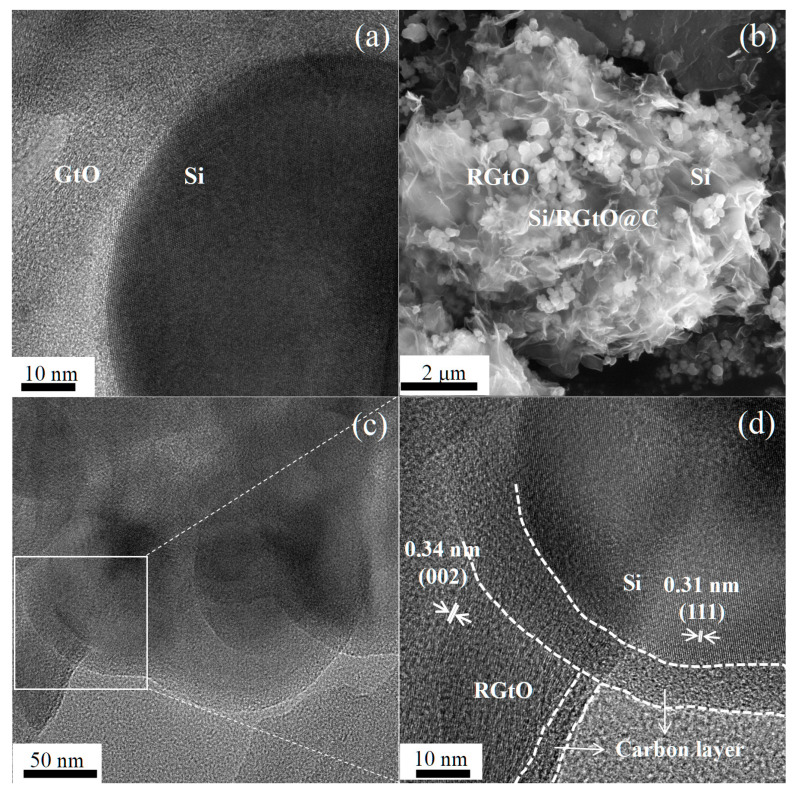
(**a**) HRTEM images of Si/GtO, (**b**) SEM image of Si/RGtO@C, (**c**,**d**) HRTEM image of Si/RGtO@C and the magnification.

Figure S1a shows the HAADF-STEM image of Si/GtO. As displayed in Figure S1b,d, the distributions of C and O elements are highly consistent, indicating abundant oxygen-containing groups in GtO. The Si nanoparticle displays no obvious outer layer (Figure 2a). In contrast, the Si nanoparticle of Si/RGtO@C composite shows a clear outer carbon layer, as depicted in Figure 2c,d. Figure 2d shows the lattice spacing of 0.34 nm, which is assigned to the graphite’s (002) plane, demonstrating the reduction of GtO. Additionally, a lattice spacing of 0.31 nm corresponding to the (111) plane of Si is observed. Due to the thin anchoring carbon layer, the morphology of the Si nanoparticles anchoring on the surface of RGtO is clearly observed, as shown in Figure 2b. In Figure S2b, the overall distribution of the Si nanoparticles on GtO is relatively uniform. In contrast, the distribution of the Si nanoparticles on RGtO are heterogeneous in the absence of the carbon coating layer (Figure S2c). As shown in Figure S3, the CTAB undergoes a significant weight loss (more than 45 wt%) at around 250 °C in an oxygen atmosphere. Due to the weight loss of CTAB by oxidization, maintaining the anchoring status of the Si nanoparticles on GtO in the thermal treatment process at 250 °C in an air atmosphere is challenging. The result indicates that the PAN coating layer plays an important role in preserving the structural stability of Si/GtO in the thermal treatment process. Compared to RGtO, the thicker carbon coating layer on Si nanoparticles (Figure 2d) confirms that the precursor PAN can be well coated on CTAB modified Si (Si@CTAB), which should be related to the electrostatic force between positively charged Si@CTAB and PAN with a large number of negatively charged groups.

**Figure 3:**
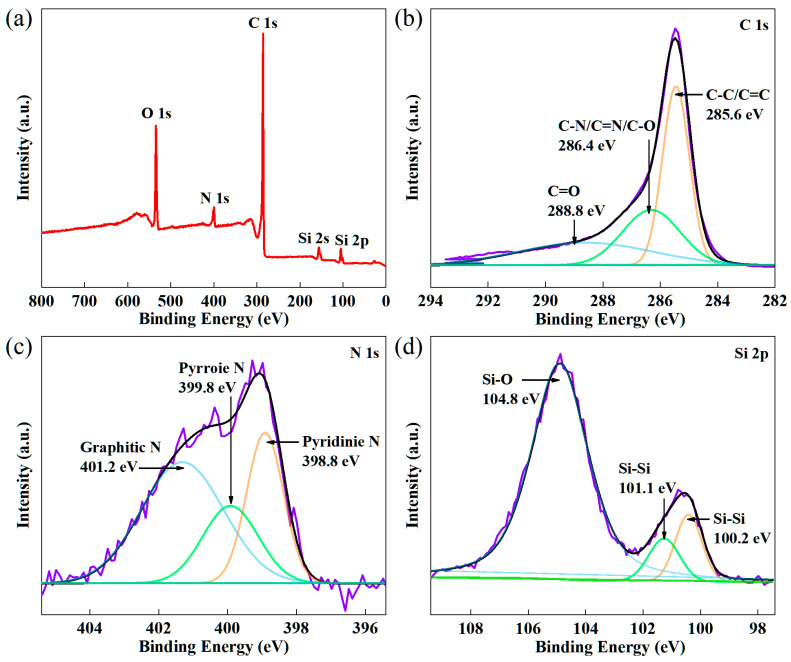
(**a**) XPS survey spectrum of Si/RGtO@C, high-resolution (**b**) C 1s, (**c**) N 1s, and (**d**) Si 2p spectrum of Si/RGtO@C.

Figure 3a displays the XPS spectrum of Si/RGtO@C, which indicates the presence of C, N, O, and Si elements in the composite. The Fermi level serves as a benchmark, and the binding energy of core-level electrons for the C 1s peak is labeled as $E_{B}^{F}$. Notably, the $E_{B}^{F}$ position is closely correlated with the work function of samples (*Φ*_SA_) of the substrate [22,23]. And the C 1s peak value of indefinite carbon is calibrated using the following formula [24]: $E_{B}^{F}$ = 289.58 − *Φ*_SA_. The *Φ*_SA_ of the Si substrate is referenced from a published value of 4.01 eV to adjust the C 1s peak value [24]; thus, the $E_{B}^{F}$ of C 1s’ peak position is 285.57 eV. Figure 3b represents a high-resolution C 1s spectrum, which is deconvoluted into three distinct peaks. The spectral signatures centered around 285.6 eV, 286.4 eV, and 288.8 eV are assigned, respectively, to the C–C/C=C bonds, C–O/C–N/C=N bonds, and C=O bonds [25,26]. The prominent peak observed near 400.0 eV corresponds to the N 1s originating from the PAN (raw material). As shown in Figure 3c, the N element consists of pyridine N (398.7 eV), pyrrole N (399.7 eV), and graphitic N (401.1 eV) [27,28]. Figure 3d depicts the high-resolution Si 2p spectrum. The peak at 100.2 eV corresponds to the Si 2p3/2 core level, while the peak at 101.1 eV is assigned to the Si 2p1/2 core level, both belonging to the Si–Si bond [29]. The peak centered at 104.8 eV is attributed to the Si–O bonds [30], which should be from SiO_2_ on the surface of the Si nanoparticles due to a slight surface oxidation of Si in the air [28,31,32]. The O 1s peak at ~532.0 eV mainly originates from the Si–O bond.

**Figure 4:**
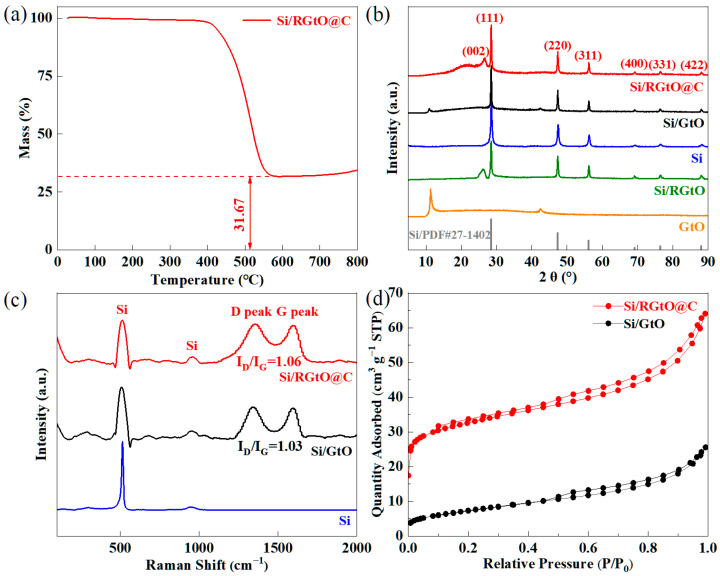
(**a**) TGA curve of the Si/RGtO@C composite in O_2_ atmosphere. (**b**) XRD patterns of Si/RGtO@C, Si/GtO, Si, Si/RGtO, and GtO. (**c**) Raman spectroscopic curves of Si/RGtO@C, Si/GtO, and Si. (**d**) N_2_ adsorption/desorption isotherms of Si/RGtO@C, and Si/GtO.

Figure 4a shows the TGA curve of Si/RGtO@C in an oxygen atmosphere. Under high temperature in an O_2_ atmosphere, both RGtO and the carbon layer are transformed into gaseous carbon oxides. The mass ratio of Si in Si/RGtO@C is approximately 31.67 wt%. There is an increase in mass after 600 °C, which results from the oxidation of Si. The XRD patterns of Si/RGtO@C, Si/GtO, Si, and GtO are shown in Figure 4b. The peaks centered at ~26.5° for Si/RGtO@C correspond to the (002) lattice plane of the graphite (PDF# 41-1487) [33]. The broad peak centered at 22.9° is attributed to the amorphous carbon of the carbon coating layer. The peak of Si/RGtO at ~26.3° corresponds to the (002) lattice plane of graphite (PDF# 41-1487). The characteristic diffraction peaks at about 28.2°, 47.3°, 56.2°, 69.2°, 76.4°, and 88.2° are assigned to the (111), (220), (311), (400), (331), and (422) lattice planes of cubic Si (PDF# 27-1402), respectively. The Raman spectra of Si/RGtO@C, Si/GtO, and Si are shown in Figure 4c. The peak located at approximately 521 cm^−1^ corresponds to the first-order transverse optical mode of Si [34]. The distinctive peaks observed at approximately 948 cm^−1^ and 293 cm^−1^ are attributed, respectively, to the second-order optical phonon and transverse acoustic phonon modes of Si [34]. Both Si/RGtO@C and Si/GtO show the characteristic D peak at about 1355 cm^−1^ and G peak at about 1598 cm^−1^. The intensity ratio of D peak to G peak (I_D_/I_G_) reflects the level of disorder of the carbon materials. The higher value indicates the lower graphitization degree [35,36]. The thermal reduction of GtO may partially damage the structure of sp^2^ C and the carbon coating layer has an amorphous structure. Those two aspects lead to a higher I_D_/I_G_ value of Si/RGtO@C (1.06) compared to that of Si/GtO (1.03) (Figure 4c). Figure 4d displays the N_2_ adsorption/desorption curves of both Si/RGtO@C and Si/GtO. The results reveal that Si/RGtO@C exhibits a specific surface area of 119.8 m^2^ g^−1^, while Si/GtO exhibits a lower specific surface area of 26.2 m^2^ g^−1^. On the one hand, the grinding reduces the diameter of flaky Si/RGtO@C compared to Si/RGtO, as shown in Figure S4. On the other hand, the oxidizations of CTAB (Figure S3) and GtO (Figure S5) at 250 °C in an air atmosphere, followed by the further reduction of GtO at 800 °C in an Ar atmosphere, should produce numerous pores. In Figure S6, the pore size distribution profiles of Si/RGtO@C and Si/GtO indicate that the higher surface area of Si/RGtO@C is mainly from micropores, especially at a pore diameter of approximately 1.2 nm.

### 2.2. Electrochemical Properties

**Figure 5:**
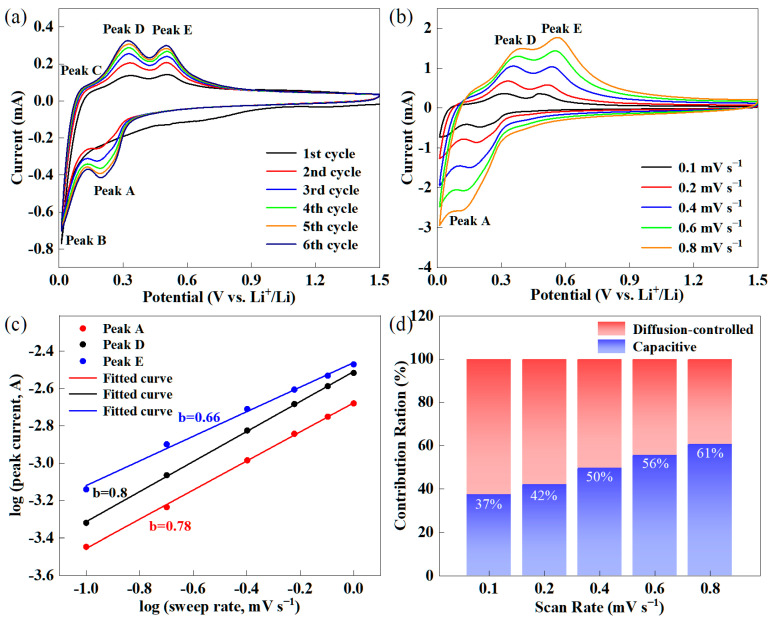
(**a**) CV profiles of Si/RGtO@C with a potential range from 0.01 to 1.5 V (vs. Li^+^ /Li) at 0.1 mV s^−1^, (**b**) CV curves of Si/RGO@C under different sweep rates, (**c**) the linear fitting of *b* under different scan rates, (**d**) the contribution rates of capacitance under different scan rates.

The cyclic voltammetry (CV) curves of Si/RGtO@C are depicted in Figure 5a. A broad peak ranging from ~1.0 to ~0.3 V is observed during the initial cathodic scanning, which is attributed to the decomposition of electrolyte and the accompanying formation of SEI film [36,37]. The cathodic peak at approximately 0.01 V (peak B) corresponds to the embedding of lithium ions into RGtO, while the anodic peak at around 0.1 V (peak C) signifies the deintercalation of lithium ions from RGtO. The anodic peaks at 0.33 and 0.51 V are due to the dealloying of Li*_x_*Si alloys [38]. The cathodic peak in the range of 0.2 to 0.3 V (peak A) is related to the lithiation of Si to form Li*_x_*Si alloy [39]. The CV curves show a gradual increase in area until the sixth cycle, indicating the gradual activation of Si/RGtO@C.

Figure 5b shows the CV curves of Si/RGtO@C across the scan rates between 0.1 and 0.8 mV s^−1^, which are used to study the electrochemical reaction kinetics of Si/RGtO@C. In general, the peak current (*i*) is correlated with the scan rate (*v*) as described by the following equation [40]:

*i* = *av^b^* (1)

log(*i*) = *b*log(*v*) + log(*a*) (2)

Generally, a *b* value of 0.5 implies that the redox process is controlled primarily by diffusion mechanisms, while a *b* value of 1.0 suggests the redox reaction is a capacitance-controlled behavior [41]. As shown in Figure 5c, the respective *b* values corresponding to peaks A, D, and E are 0.78, 0.80, and 0.66, suggesting a combined electrochemical kinetics behavior with both diffusion- and capacitance-controlled processes contributing. Furthermore, the relationship between capacitance contribution and diffusion-controlled contribution can be expressed by the following equation [40]:

*i*(*v*) = k_1_*v* + k_2_*v*^1/2^ (3)

For convenience in analysis, Equation (3) is rearranged as follows:

*i*(*v*)/*v*^1/2^ = k_1_*v*^1/2^ + k_2_ (4)

In Equation (4), the total current is a combination of a surface-confined current (k_1_*v*) and a bulk diffusion current (k_2_*v*^1/2^) [42]. To obtain the values of k_1_ and k_2_, the *i*(*v*)/*v*^1/2^ and *v*^1/2^ in Equation (4) are linearly fitted. As shown in Figure 5d, the capacitive contribution ratios increase with the scan rates because the capacitive process has fast charging/discharging characteristics [42]. The percentages of capacitive contributions progressively rise from 37% to 61% as the scan rates increase from 0.1 to 0.8 mV s^−1^. The lithium-ion storage kinetics of Si/RGtO@C are governed by a diffusion process at slow scan rates. Conversely, a capacitive controlled process is dominant at high scan rates [43]. The conversion in dominant control is consistent with findings reported for the Si/graphite anodes [43,44]. The capacitive contribution based on the CV curve at 0.8 mV s^−1^ is shown in Figure S7a.

The Nyquist plot for Si/RGtO@C exhibits a semicircle in the high-frequency domain and a line in the low-frequency domain (Figure S7b). The intercept on the Z′ axis represents the electrolyte resistance (*R*s). The diameter of the semicircle is assigned to the charge transfer resistance (*R*ct), while the sloping line signifies the Warburg impedance (*W*). The Nyquist plots of the impedance spectra show that the *R*ct value of Si/RGtO@C decreases from around 75 Ω to around 10 Ω after the initial cycle at 1.0 A g^−1^, indicating the electrode is activated during the cycle. The equivalent circuit includes *R*s, *R*ct, constant phase element (CPE), and *W*. The *R*ct of Si/RGtO@C is significantly lower than that of Si/GtO (Figure S8), suggesting that the carbon coating layer and thermal reduction of GtO greatly enhance the overall electronic conductivity [45]. After the first charge/discharge cycle, the slope of the line for Si/RGtO@C increases slightly, indicating a faster lithium-ion diffusion behavior [46].

**Figure 6:**
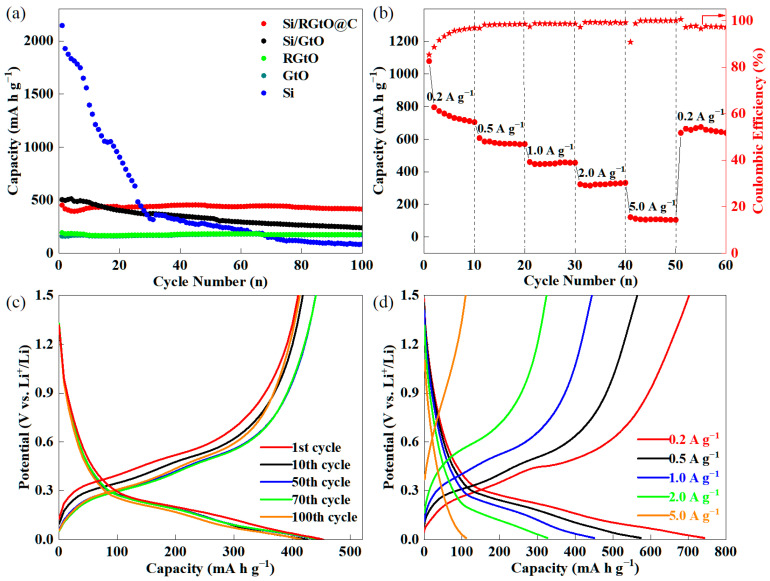
(**a**) Cycling performance of Si/RGtO@C, Si/GtO, RGtO, GtO, and Si at 1.0 A g^−1^, (**b**) discharge capacity and corresponding Coulombic efficiency of Si/RGtO@C under different current densities (the dots are the discharge capacities under different current densities and the asterisks are the corresponding Coulombic efficiencies), (**c**) charge/discharge characteristics of Si/RGtO@C at 1.0 A g^−1^ over different cycling stages, (**d**) specific charge and discharge profiles of Si/RGtO@C under different current densities.

**Figure 7:**
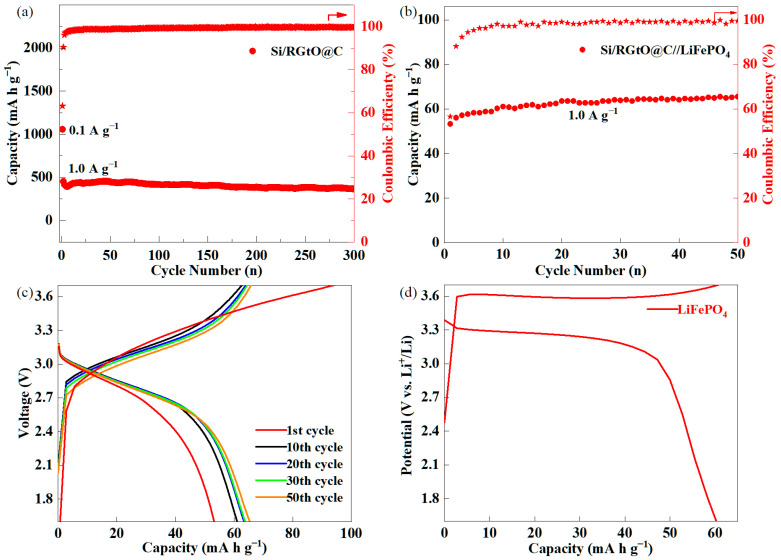
(**a**) Long-cycle capacity and Coulombic efficiency of Si/RGtO@C at 1.0 A g^−1^, (**b**) cycling performance of Si/RGtO@C//LiFePO_4_ full cell at 1.0 A g^−1^ within a voltage range of 1.6–3.7 V (the dots are the discharge capacities at 1.0 A g^−1^ at different cycle numbers and the asterisks are the corresponding Coulombic efficiencies), (**c**) charge/discharge curves of Si/RGtO@C//LiFePO_4_ full cell at 1.0 A g^−1^ over different cycles, (**d**) representative charge/discharge curves of LiFePO_4_ in Li//LiFePO_4_ half cell at 1.0 A g^−1^ within a potential range of 1.6–3.7 V.

Figure 6a illustrates the cycling performance of Si/RGtO@C, Si/GtO, RGtO, GtO, and Si at 1.0 A g^−1^. Initially, a small current density of 0.1 A g^−1^ was utilized to build a stable SEI film. The Coulombic efficiency of Si/RGtO@C in the first cycle at 0.1 A g^−1^ was about 63.1%, and the detailed charge/discharge curves are exhibited in Figure S9. After 100 cycles, the capacities of Si/RGtO@C, Si/GtO, RGtO, GtO, and Si were 416.8, 239.5, 175.1, 174.0, and 86.1 mA h g^−1^, respectively. Notably, the specific capacity of Si/RGtO@C surpassed the combined specific capacity of Si and RGtO. The Si/RGtO@C retains 91.9% of its initial capacity (453.5 mA h g^−1^ at 1.0 A g^−1^) after 100 cycles, markedly outperforming Si/GtO. The charge/discharge profiles of Si/RGtO@C at 1.0 A g^−1^ under the 1st, 10th, 50th, 70th, and 100th cycles are illustrated in Figure 6c. The above charge/discharge profiles sequentially correspond the discharge capacities of 453.5, 426.4, 445.5, 445.8, and 416.8 mA h g^−1^, demonstrating high capacity and good cycling stability. During the charge and discharge process, the RGtO and carbon layer not only effectively mitigate the volume changes but also serve to compensate for the poor electronic conductivity of the Si nanoparticles. As represented in Figure 6b, the Si/RGtO@C anode exhibits capacities of 795.6, 610.2, 459.1, 322.7, and 120.8 mAh g^−1^ at the current densities of 0.2, 0.5, 1.0, 2.0, and 5.0 A g^−1^, respectively. While returning to 0.2 A g^−1^, the Si/RGtO@C anode maintains a discharge capacity of 638.8 mAh g^−1^. Figure 6d displays the detailed charge/discharge curves of Si/RGtO@C at different current densities. The potential difference between the charging and discharging curve gradually increases with the increase in current density which is caused by polarization. As shown in Figure 7a, after an initial cycle at 0.1 A g^−1^, Si/RGtO@C maintains a stable capacity and high Coulombic efficiency over the long cycles. After 300 cycles at 1.0 A g^−1^, Si/RGtO@C still retains a high specific discharge capacity of 367.6 mA h g^−1^ and a high Coulombic efficiency of approximately 99.8%. The average capacity decay rate of Si/RGtO@C at 1.0 A g^−1^ is only 0.063% per cycle. The excellent electrochemical performance is mainly due to the evidence that the carbon materials can compensate for the insufficient conductivity of Si and accommodate the volume changes of Si during charge-discharge processes, as reported in the literature [36,39,47]. Furthermore, the electrochemical performance of the Si/RGtO@C anode is comparable to previously reported Si/C composites [20,43,47,48,49,50,51,52,53]. Detailed information is provided in Table S1. Before assembling the full cell, the Si/RGtO@C anode was cycled at 0.1 A g^−1^ for one cycle and then at 1.0 A g^−1^ for the next five cycles to build a stable SEI film and recompense the irreversible lithium loss in the first cycle of full cell [54,55,56]. Figure 7b shows the cycling performance of the Si/RGtO@C//LiFePO_4_ full cell at 1.0 A g^−1^. After 50 cycles, the LiFePO_4_ cathode still maintains a capacity of 65.5 mA h g^−1^ and a high Coulombic efficiency of approximately 99.5%. The charge/discharge curves of the Si/RGtO@C//LiFePO_4_ full cell at 1.0 A g^−1^ over different cycles are shown in Figure 7c. The discharge capacities of LiFePO_4_ at the 1st, 10th, 20th, 30th, and 50th cycles are 53.3, 61.1, 63.6, 63.9, and 65.5 mA h g^−1^, respectively, indicating high capacities and stable cycling performance. Figure 7d displays representative charge/discharge curves of LiFePO_4_ in the Li//LiFePO_4_ half cell at 1.0 A g^−1^ within a potential range of 1.6–3.7 V. As can be seen, the discharge capacity of LiFePO_4_ in the half cell is about 60.5 mA h g^−1^. The findings suggest that the Si/RGtO@C anode holds the promise of potential for practical application in high-energy-density lithium-ion batteries.

## 3. Materials and Methods

### 3.1. Material Preparation

A total of 0.25 g of Si nanoparticles (50 nm, New Iron & Metal Materials Co., Ltd., Xingtai, China) was put into 30 mL of 0.01 M aqueous solution of CTAB (AR, Sinopharm Chemical Reagent Co., Ltd., Beijing, China) and sonicated for 30 min. The mixture was loaded into a 50 mL Teflon-lined stainless-steel autoclave and subjected to heat treatment at 120 °C for 2 h. The positively charged Si (Si@CTAB) nanoparticles were obtained by centrifugation, washing, and freeze-drying. The Si@CTAB nanoparticles were subsequently mixed with a GtO aqueous dispersion (GtO-3, 99.5%, Hangzhou Gaoene Technology Co., Ltd., Hangzhou, China) in a mass ratio of 1:3 (Si:GtO). The mixture was allowed to stand for 1 h, during which the Si/GtO composite assembled through electrostatic forces. Finally, the Si/GtO composite was obtained after centrifugation, washing, and freeze-drying.

PAN with a molecular weight of 150,000 (J&K Scientific Ltd., Beijing, China) was dissolved in 15 mL of N, N-dimethylformamide (DMF, AR, Sinopharm Group) through stirring at 70 °C for 6 h. Subsequently, the Si/GtO was incorporated into the solution, maintaining a PAN-to-Si/GtO mass ratio of 1:3. After stirring for 2 h, the resulting paste was uniformly coated onto a nickel foil and subsequently underwent oven drying at 60 °C for 24 h. And then the Si/GtO@PAN flakes were detached from the nickel foil. To pre-oxidize the PAN, Si/GtO@PAN underwent a thermal treatment at 250 °C for 2 h with a heating rate of 1 °C/min. Finally, Si/RGtO@C was fabricated after the further thermal treatment at 800 °C for 2 h under an argon atmosphere.

For comparison, RGtO was prepared from GtO using the same thermal treatment process. Similarly, the Si/RGtO composite was obtained in the absence of PAN.

### 3.2. Material Characterization

To investigate the mass fraction of Si, thermogravimetric analysis (TGA) was undertaken utilizing the NETZSCH STA449F5 thermogravimetric analyzer (Selb, Germany) in an O_2_ environment. The crystallographic features of the specimens were characterized via the Bruker D8 Advance powder X-ray diffraction (XRD) process analyzer (Karlsruhe, Germany) employing Cu-Kα radiation as X-ray source with a wavelength of 1.5418 Å. The elemental compositions of samples without sputter etching were gained through X-ray photoelectron spectroscopy (XPS) measurements on the Thermo Fisher Scientific ESCALAB 250Xi system (Waltham, MA, USA)**.** The structural properties of samples were observed by the Thermo Fisher Scientific DXR2 laser Raman spectrometer (Waltham, MA, USA) with an excitation wavelength of 532 nanometers. To assess the surface area properties, N_2_ adsorption and desorption isotherms were measured on the Micromeritics ASAP 2020 HD88 analyzer (Micromeritics, Norcross, GA, USA), and the specific surface areas were derived through the Brunauer–Emmett–Teller (BET) methodology. The microstructural morphology was characterized using both scanning electron microscopy (SEM) (Apre S HiVac, FEI, Waltham, MA, USA) and field emission transmission electron microscopy (TEM) (Talos F200S, FEI, Waltham, MA, USA). The elemental mappings of the carbon (C), nitrogen (N), and Si of the samples were performed to precisely observe their distributions.

### 3.3. Electrochemical Tests

Pulverized active material (Si/RGtO@C, Si/GtO, Si, RGtO, or GtO) was mixed with Ketjen black (EC-300J, Lion Specialty Chemicals Ltd., Tokyo, Japan) and sodium alginate (biochemical grade, Aladdin) in a precise mass ratio of 8:1:1, using deionized water to adjust the viscosity, resulting in a uniform slurry. And then the slurry was coated on copper foil. After drying, the electrode film was rolled and punched. The mass loading of active material in each disc was approximately 1.1 mg. Coin-type CR2032 cells were assembled in an Ar-filled glove box, utilizing Li metal foil as the counter electrode, a Celgard 2400 membrane as the separator, and 1 M LiPF_6_ in a 1:1 volume ratio of ethylene carbonate (EC) to diethyl carbonate (DEC) with 5 vol.% fluoroethylene carbonate (FEC) as the electrolyte. Galvanostatic charge/discharge tests were conducted on a CT-3008W battery test system (Xinwei, Shenzhen, China) in a potential range from 1.5 to 0.01 V (vs. Li^+^/Li). The specific capacity was determined based on the overall mass of the active material. CV measurements were performed on a PARSTAT MC multichannel potentiostat (Princeton Applied Research, Oak Ridge, TN, USA). Electrochemical impedance spectroscopy (EIS) measurements were conducted using a voltage perturbation of 5 mV over a frequency sweep ranging from 100 kHz to 0.1 Hz.

Additionally, full coin cells were assembled using Si/RGtO@C as the anodic active material and commercial LiFePO_4_ (98%, Aladdin) as the cathodic active material. The galvanostatic charge/discharge capacity of the full cell was measured at 1.0 A g^−1^ within a voltage range between 1.6 and 3.7 V, and the specific capacity was calculated based on LiFePO_4_. And Li//LiFePO_4_ half coin cells were also assembled to compare the specific discharge capacity of LiFePO_4_.

## 4. Conclusions

In summary, the Si/RGtO@C composite was successfully prepared through electrostatic self-assembly followed by thermal treatment. This composite demonstrated a significantly higher specific capacity compared to Si and RGtO individually. After 300 cycles of charge and discharge at 1.0 A g^−1^, Si/RGtO@C kept a capacity of 367.6 mA h g^−1^ and a high capacity retention of 81.1%. The carbon coating layer played a crucial role in improving the interface contact between the Si nanoparticles and RGtO, thereby enhancing the structural stability of Si/RGtO@C during charge/discharge cycling. Even after 300 charge/discharge cycles at 1.0 A g^−1^, Si/RGtO@C still maintained a capacity of 367.6 mA h g^−1^, with a capacity retention of 81.1%. The carbon coating layer played a crucial role by improving the contact between the Si nanoparticles and RGtO, enhancing the structural stability of Si/RGtO during charge/discharge cycles. The RGtO’s synergistic carbon coating layer promoted the electrochemical performance of the Si nanoparticles, making Si/RGtO@C a high-performance anode material. Based on the exceptional electrochemical performance and a fabrication process amenable to large-scale production, this research provides insights into the design and development of the Si/C-based anode materials aimed at advancing battery technology.

## Data Availability

The data presented in this study are available in article and Supplementary Materials.

## Supplementary Materials

The following supporting information can be downloaded at: https://www.mdpi.com/article/10.3390/molecules29174108/s1, Figure S1: (a) HAADF-STEM image of Si/GtO and the elemental mappings of (b) C, (c) Si and (d) O; Figure S2: SEM images of (a) Si nanoparticles and (b) Si/GtO composite; Figure S3: TGA curve of CTAB in an oxygen atmosphere; Figure S4: SEM images of (a) Si/GtO and (b) Si/RGtO@C composite; Figure S5: TGA curve of GtO in an oxygen atmosphere; Figure S6: Pore-size distribution curves of Si/RGtO@C and Si/GtO; Figure S7: (a) CV profile with capacitive contribution at a scan rate of 0.8 mV s^−1^, (b) Nyquist plots for Si/RGtO@C before and after the first charge-discharge cycle at 1.0 A g^−1^ and the equivalent circuit modeling for the electrochemical impedance spectroscopy; Figure S8: (a) Nyquist plots of fresh Si/RGtO@C and Si/GtO electrode, (b) Nyquist plots of Si/RGtO@C and Si/GtO electrode after the initial charge/discharge cycle at 1.0 A g^−1^; Figure S9: The charge/discharge curves of Si/RGtO@C during the first cycle at 0.1 A g^−1^. Table S1: The comparison of Si/RGtO@C with other Si/C composites on lithium storage capacity.
