# Supplementary material for: Si/Graphite@C Composite Fabricated by Electrostatic Self-Assembly and Following Thermal Treatment as an Anode Material for Lithium-Ion Battery
Source: Molecules. 2024 Aug 29;29(17):4108. doi: 10.3390/molecules29174108 (PMC11397206; doi:10.3390/molecules29174108)
Supplement: Supplementary file 1 [file molecules-29-04108-s001.zip › molecules-3107506-supplementary.pdf]

Si/Graphite@C Composite Fabricated by Electrostatic Self-Assembly and following Thermal Treatment as an Anode Material for Lithium-Ion Battery

Jintao Yao [1,#], Guangzhao Zhu [1,#], Jingrui Huang [1], Xiaoru Meng [1], Maolong Hao [1,2], Shoupu Zhu [1,*], Zhen Wu [3,4,*], Fanxu Kong [2], Yue Zhou [1], Qi Li [5], Guowang Diao [6]

[1] College of Energy Storage Technology, Shandong University of Science and Technology, Qingdao 266590, China

[2] College of Electrical Engineering and Automation, Shandong University of Science and Technology, Qingdao 266590, China

[3] School of Energy and Power Engineering, Jiangsu University, Zhenjiang, 212013, China

[4] FEB Research Institute, Far East Battery, Wuxi 214200, China

[5] Suzhou Institute of Nano-Tech and Nano-Bionics, Chinese Academy of Sciences, Suzhou 215123, China

[6] School of Chemistry and Chemical Engineering, Yangzhou University, Yangzhou 225002, China

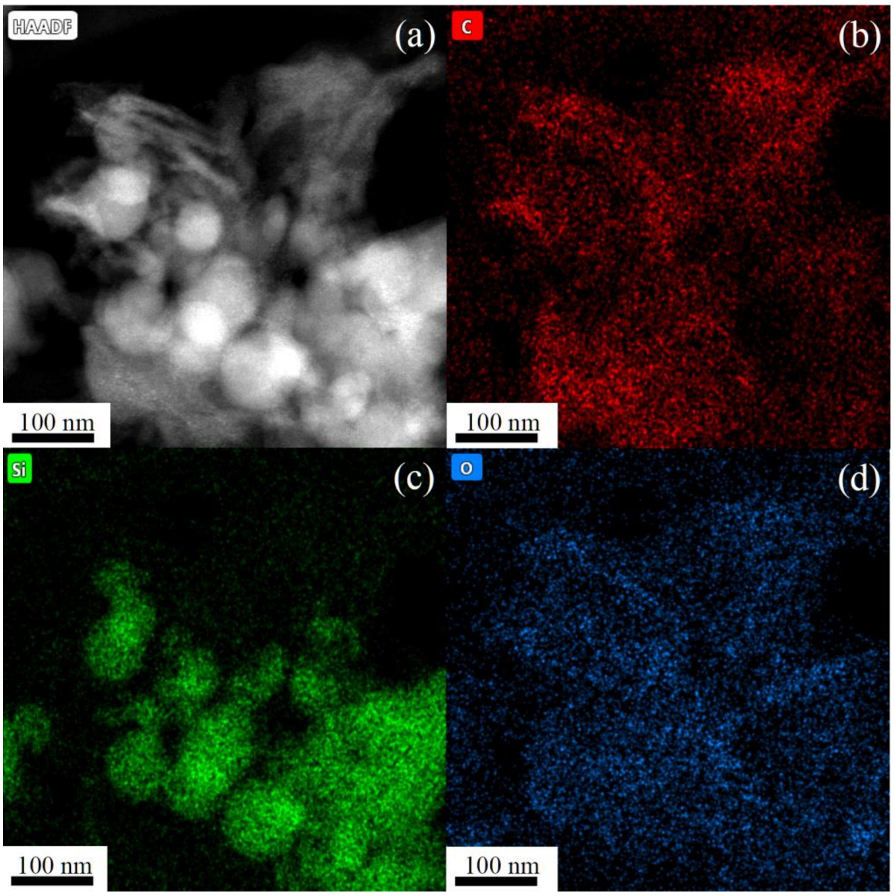

Figure S1. (a) HAADF-STEM image of Si/GtO and the elemental mappings of (b) C, (c) Si and (d) O.

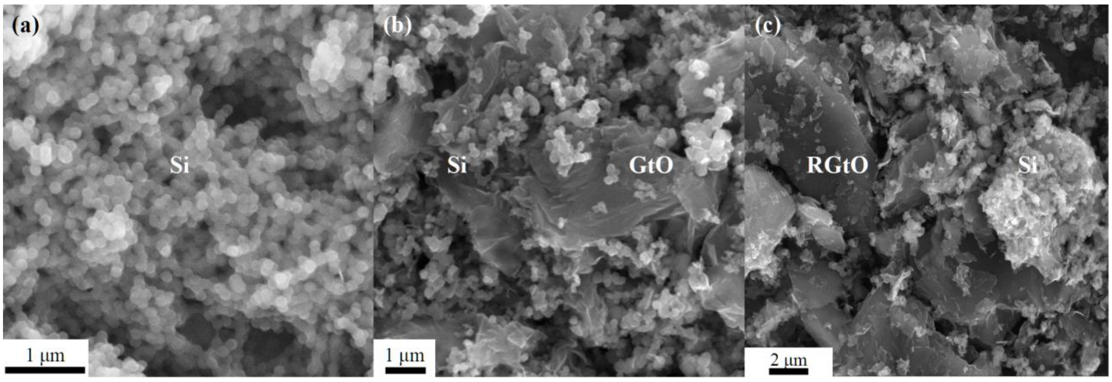

Figure S2. SEM images of (a) Si nanoparticles, (b) Si/GtO and (c) Si/RGtO composite.

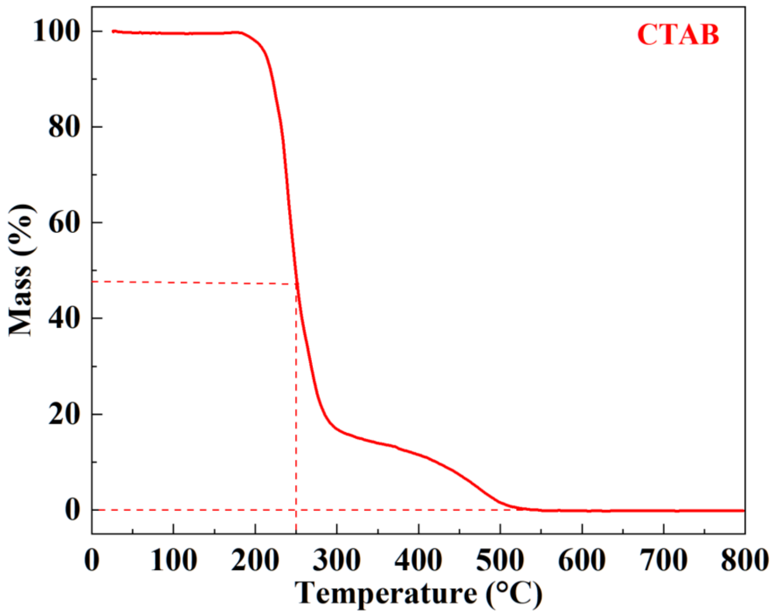

Figure S3. TGA curve of CTAB in an oxygen atmosphere.

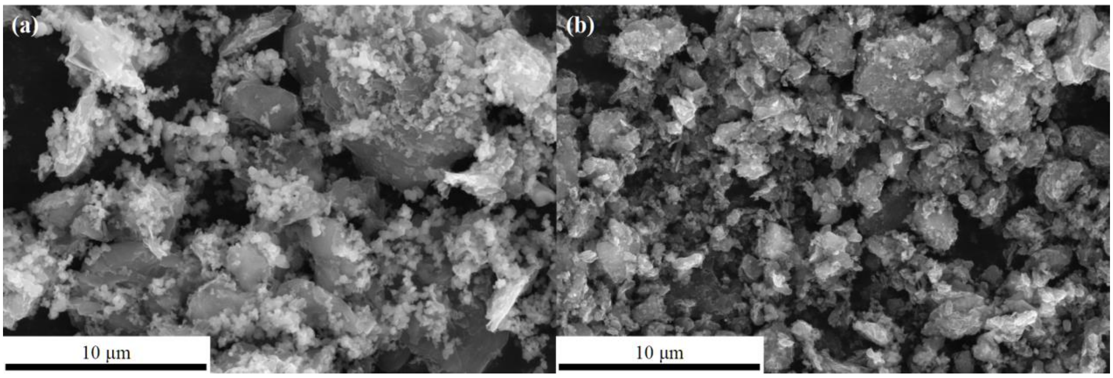

Figure S4. SEM images of (a) Si/GtO and (b) Si/RGtO@C composite.

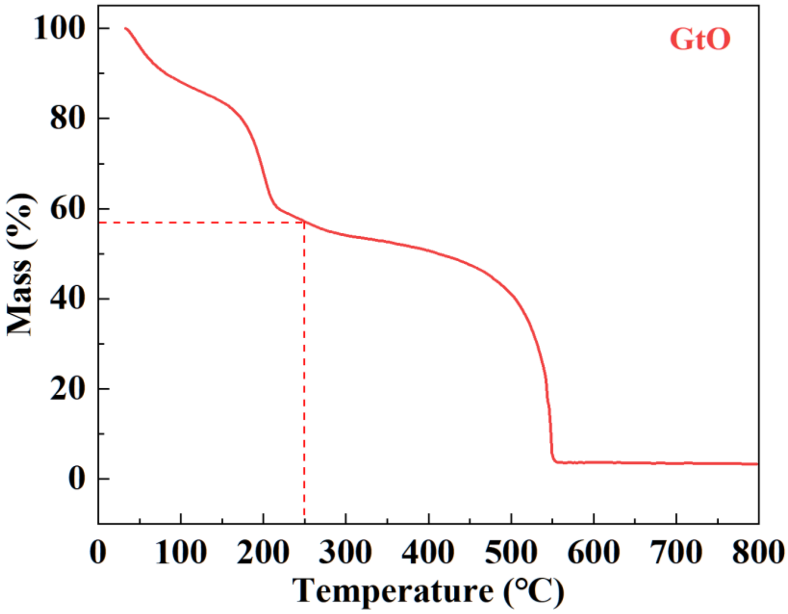

Figure S5. TGA curve of GtO in an oxygen atmosphere.

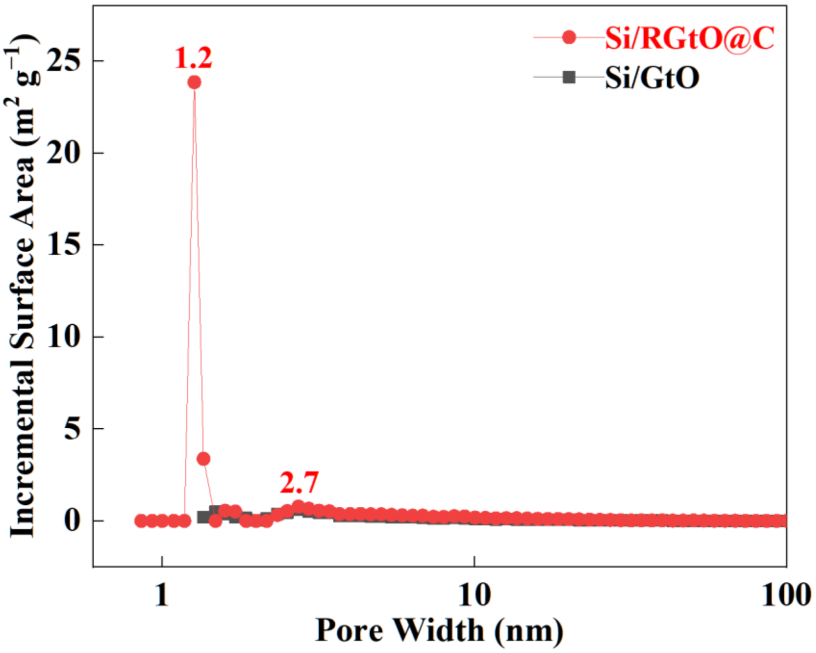

Figure S6. Pore-size distribution curves of Si/RGtO@C and Si/GtO.

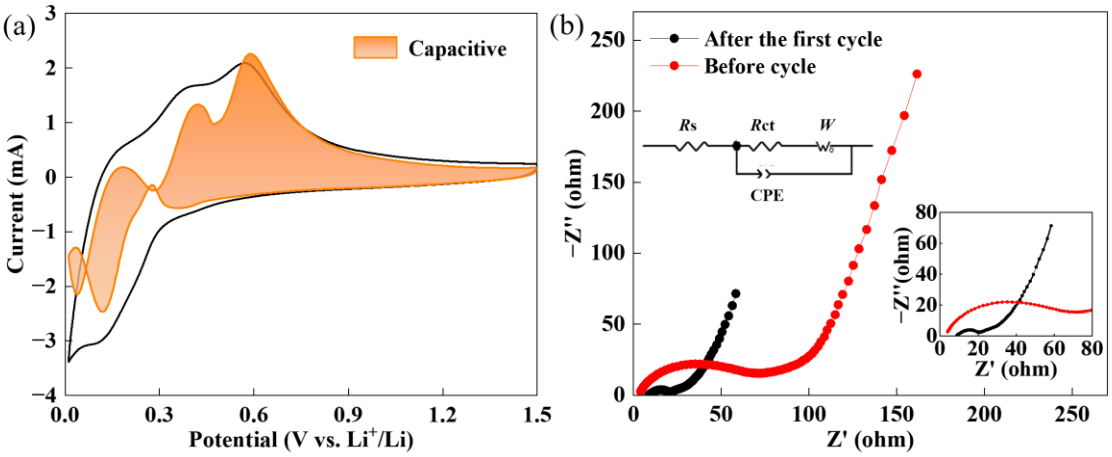

Figure S7. (a) CV profile with capacitive contribution at a scan rate of 0.8 mV s^{-1}, (b) Nyquist plots for Si/RGtO@C before and after the first charge-discharge cycle at 1.0 A g^{-1} and the equivalent circuit modeling for the electrochemical impedance spectroscopy.

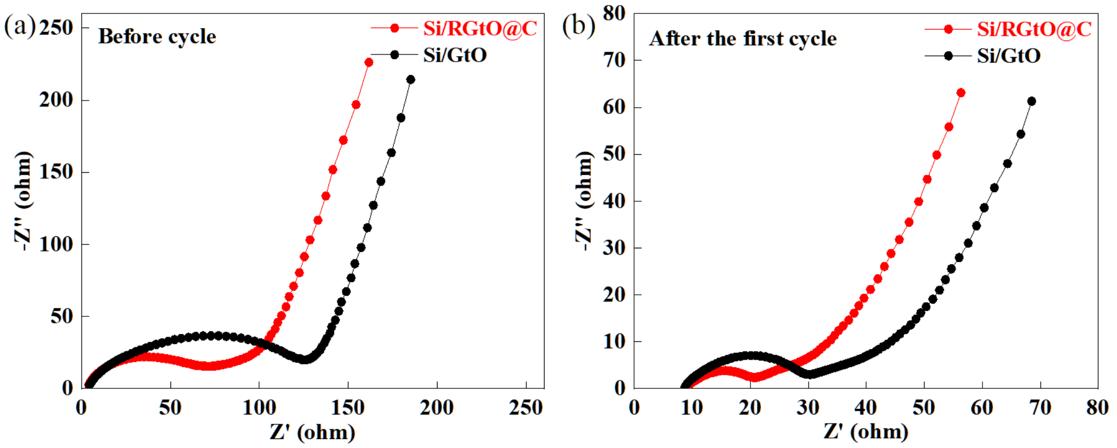

Figure S8. (a) Nyquist plots of fresh Si/RGtO@C and Si/GtO electrode, (b) Nyquist plots of Si/RGtO@C and Si/GtO electrode after the first charge/discharge cycle at 1.0 A g^{-1}.

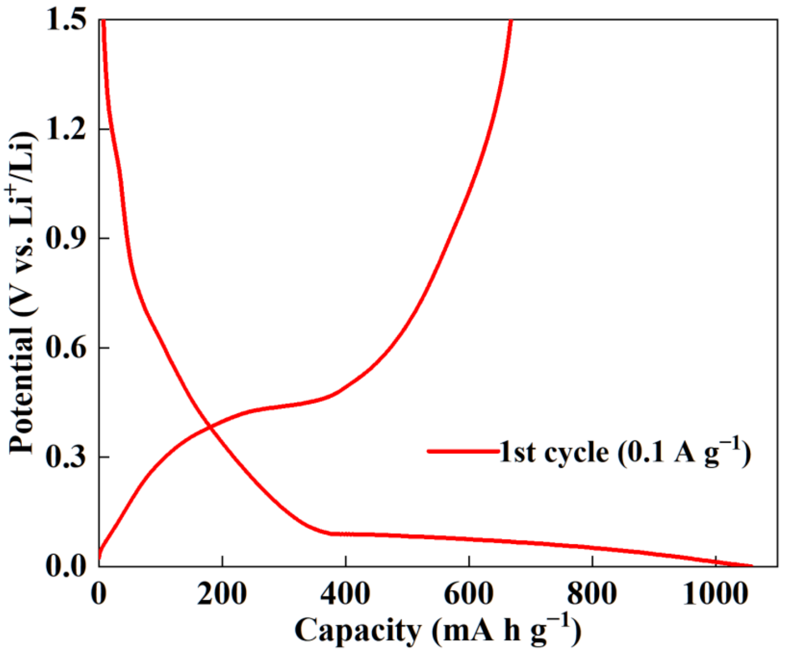

Figure S9. The charge/discharge curves of Si/RGtO@C during the first cycle at 0.1 A g^{-1}.

Table S1 The comparison of Si/RGtO@C with other Si/C composites on lithium storage capacity.

Si/C anodes	Current density (mA g^{-1})	Cycles	Capacity (mAh g^{-1})	Reference
Si/RGtO@C	1000	300	368	This work
Si@C/G	1000	50	592	[20]
Si&AG	1000	500	200	[43]
Si/G/C	500	200	400	[47]
Si@FG/C	500	300	420	[48]
Si/G	1000	400	485.3	[49]
Si/G/DC	168	260	420	[50]
Si/C@NGs	100	100	428	[51]
Si-Graphite	53	50	442	[52]

Si-C-Graphite	500	100	484	[53]

Note: G = graphite, FG = flake-graphite, DC = disordered carbon, NG = natural graphite, AG = artificial graphite.

References

[20] Liu, Y.; Liu, X.; Zhu, Y.; Wang, J.; Ji, W.; Liu, X. Scalable synthesis of pitch-coated nanoporous Si/graphite composite anodes for lithium-ion batteries. *Energy Fuels* **2023**, *37*, 4624–4631.

[43] Dong, Y.; Liu, C.; Li, F.; Jin, H.; Li, B.; Ding, F.; Yang, Y.; Ha, M.N.; Tran, D.L.; Yuan, F. Reinforced interfacial interaction between Si and graphite to improve the cyclic stability of lithium-ion batteries. *ACS Appl. Mater. Interfaces* **2024**, *16*, 23416–23425.

[47] Liu, W.; Zhong, Y.M.; Yang, S.Y.; Zhang, S.S.; Yu, X.Y.; Wang, H.Q.; Li, Q.Y.; Li, J.; Cai, X.; Fang, Y.P. Electrospray synthesis of nano-Si encapsulated in graphite/carbon microplates as robust anodes for high performance lithium-ion batteries. *Sustain. Energy Fuels* **2018**, *2*, 679–687.

[48] Wang, H.; Xie, J.; Zhang, S.C.; Cao, G.S.; Zhao, X.B. Scalable preparation of silicon@graphite/carbon microspheres as high-performance lithium-ion battery anode materials. *RSC Adv.* **2016**, *6*, 69882–69888.

[49] Chen, M.X.; Cao, W.Y.; Wang, L.C.; Ma, X.; Han, K. Chessboard-like silicon/graphite anodes with high cycling stability toward practical lithium-ion batteries. *ACS Appl. Energy Mater.* **2021**, *4*, 775–783.

[50] Li, M.-Q.; Qu, M.-Z.; He, X.-Y.; Yu, Z.-L. Effects of electrolytes on the electrochemical performance of Si/graphite/disordered carbon composite anode for lithium-ion batteries. *Electrochim. Acta* **2009**, *54*, 4506–4513.

[51] Wang, Z.L.; Mao, Z.M.; Lai, L.F.; Okubo, M.; Song, Y.H.; Zhou, Y.J.; Liu, X.; Huang, W. Sub-micron silicon/pyrolyzed carbon@natural graphite self-assembly composite anode material for lithium-ion batteries. *Chem. Eng. J.* **2017**, *313*, 187–196.

[52] Hou, J.; Gong, B.L.; Hou, C.P.; Wang, B.P.; Yang, D.; Wang, X.W. Facile synthesis of nano-Si modified graphite composite as anode material for lithium ion batteries. *Int. J. Electrochem. Sci.* **2019**, *14*, 3455–3464.

[53] Parekh, M.H.; Sediako, A.D.; Naseri, A.; Thomson, M.J.; Pol, V.G. In situ mechanistic elucidation of superior Si-C-Graphite Li-ion battery anode formation with thermal safety aspects. *Adv. Energy Mater.* **2020**, *10*, 1902799.
